# Leader Narcissism and Employee Organizational Citizenship Behavior Directed Toward the Leader: Roles of Perceived Insider Status and Need for Self-Esteem

**DOI:** 10.3389/fpsyg.2021.747330

**Published:** 2021-11-15

**Authors:** Huaqiang Wang, Geng Liu, Miao Wang, Yue Dong

**Affiliations:** ^1^School of Economics and Management, Yangtze University, Jingzhou, China; ^2^School of Business Administration, Zhongnan University of Economics and Law, Wuhan, China; ^3^School of International Education, Wuhan Sports University, Wuhan, China

**Keywords:** leader narcissism, perceived insider status, OCB-L, need for self-esteem, self-concept theory

## Abstract

Based on self-concept theory, the present study proposed and empirically tested the impact of leader narcissism on employee organizational citizenship behavior directed toward the leader (OCB-L), highlighting the mediating role of perceived insider status and the moderating role of need for self-esteem in this relationship. Based on an analysis of 161 two-stage matched leader-employee dyads, the hypotheses were tested and the results showed that the leader narcissism had a negative direct effect on employee OCB-L, as well as a negative indirect effect on employee OCB-L *via* perceived insider status. Furthermore, the need for self-esteem was found to moderate the negative effect of leader narcissism on perceived insider status as well as the mediating effect of perceived insider status between leader narcissism and employee OCB-L. The theoretical and practical implications of our research were discussed. Limitations and directions for future research were also offered.

## Introduction

Narcissism is a personal trait that encompasses grandiosity, self-love, arrogance, self-absorption, entitlement, and an inflated self-view (Rosenthal and Pittinsky, 2006; Campbell et al., 2011). Studies show that narcissistic individuals tend to stand out and become leaders in the fierce competition within organizations (Brunell et al., 2008; Nevicka et al., 2011; Ong et al., 2016). In recent years, some successful business “stars,” such as Steve Jobs, Elon Musk, Bill Gates, and Jack Welch, who to some extent all have narcissistic personalities, have been labeled as narcissistic leaders by the media (Visser et al., 2017). In light of this, leader narcissism has gradually become a hot topic in the field of organizational research and has been an issue of wide concern in the academic community (O’Boyle et al., 2012; Owens et al., 2015).

Given that leader narcissism has been shown to substantially impact employees (O’Boyle et al., 2012), there is growing interest in identifying its specific effects. Prior studies have focused on the effects of leader narcissism on employees’ job performance (Owens et al., 2015; Liu et al., 2021) and workplace behaviors such as voice behavior (Liao et al., 2019b; Huang et al., 2020; Yao et al., 2020), innovation behavior (Yang et al., 2020; Norouzinik et al., 2021), change-oriented organizational citizenship behavior (Ha et al., 2020), and counterproductive behavior (O’Boyle et al., 2012; Braun et al., 2018). Although existing studies have provided valuable insights into the ways in which leader narcissism affects employees’ individual-directed and organization-directed behaviors, it does not answer the question of how leader narcissism affects employees’ leader-directed followership behavior. Organizational citizenship behavior directed toward leaders (OCB-L) refers to a discretionary extra-role behavior aimed at benefiting the leaders, which includes helping leaders with their work and accepting extra duties and responsibilities at work (Williams and Anderson, 1991; Liao and Rupp, 2005; Li et al., 2018). This kind of behavior can meet the unique needs of narcissistic leaders (Morf and Rhodewalt, 2001), and this can not only effectively promote their leadership but can also enable employees to obtain better work support (Allen and Rush, 1998). In this fashion, it forms a benign interaction between leaders and employees (Li et al., 2018). However, there is still an inadequacy of information on the relationship between leader narcissism and employee OCB-L.

Our study draws from self-concept theory (Shamir et al., 1993) and the narcissism literature to propose a moderated mediation model that delineates how and under what conditions leader narcissism affects employee OCB-L. According to self-concept theory, employees keep checking and verifying the part that is consistent with their self-concept (including self-conception and self-evaluation) in their interactions with leaders and exhibit behaviors consistent with that self-concept (Shamir et al., 1993; Sun et al., 2014). Perceived insider status, the self-conception dimension of self-concept (Chen and Aryee, 2007), refers to the extent to which an individual perceives himself or herself to be an insider (Dai and Chen, 2015). Relevant studies have shown that leader behaviors and leadership style have a strong influence on employees’ perceived insider status (Chen and Aryee, 2007; Liao, 2015; Schaubroeck et al., 2017), which in turn affects employees’ organizational citizenship behaviors (OCBs; Podsakoff et al., 2000; Stamper and Masterson, 2002). Thus, we propose that leader narcissism may threaten employees’ sense of value and acceptance at work, so that employees’ perceived insider status will be weakened, leading to a series of negative employee behaviors such as lessened OCB-L.

In addition, the need for self-esteem measures an individual’s need for positive evaluation such as their leaders’ affirmation and praise (Hill, 1987). To some extent, the need for self-esteem is an embodiment of feelings of self-worth and affects the extent to which employees tie their self-concept to their leaders or outsiders (Hill, 1987; Armeli et al., 1998). Compared to employees with a low need for self-esteem, employees with a high need for self-esteem are more eager to receive positive feedback from their leaders because their feelings of self-worth are strongly dependent on the outsiders’ positive evaluations and have stronger cognitive and behavioral responses to negative evaluations. This strengthens the negative impact of leader narcissism (Fuller et al., 2006; Shen et al., 2009). Therefore, this study introduces employees’ need for self-esteem as a contingent factor in order to examine the utility boundary of leader narcissism.

**Figure 1:**
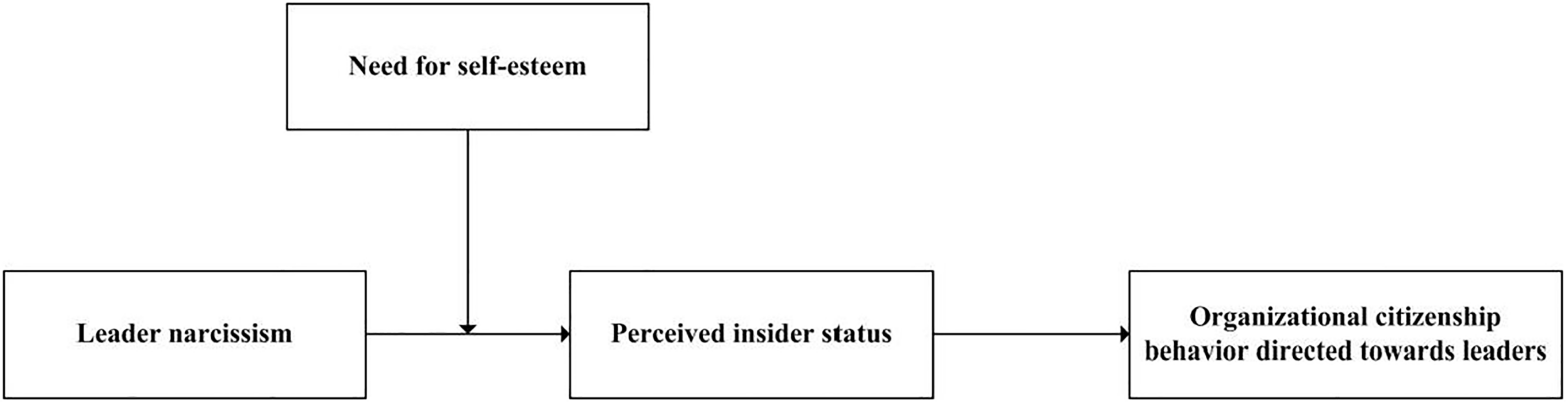
Theoretical model.

Our theoretical model contributes to the existing literature in the following ways. First, based on self-concept theory, our study analyzes the relationship between leader narcissism and employee OCB-L. This not only provides an important supplement to prior studies on the relationship between leader narcissism and employee followership behavior but also responds to researchers’ appeal to explore leader narcissism from multiple theoretical perspectives (Huang et al., 2020). Second, our study clarifies how leader narcissism affects OCB-L by revealing the mediating role of perceived insider status. This enriches our knowledge of the internal mechanism by which leader narcissism affects employee OCB-L from the perspective of self-conception. Finally, by examining the moderating role of the need for self-esteem on the relationship between leader narcissism and employee OCB-L, our research contributes to deepen understanding of the contingency factors that influence the outcomes of leader narcissism. Figure 1 presents our theoretical model, which we will explain in more detail in the next sections.

## Theory and Hypotheses

### Leader Narcissism and Employee OCB-L

The term narcissism refers to a personality trait encompassing grandiosity, arrogance, self-absorption, entitlement, fragile self-esteem, and hostility (Rosenthal and Pittinsky, 2006). Narcissistic leaders tend to take credit for their employees’ achievements and are more likely to misjudge when evaluating employees’ performance (Aquino et al., 2006). As a result, subordinates of narcissistic leaders often feel unfairly treated and even deprived because their personal interests and achievements are not guaranteed. Narcissistic leaders often resort to manipulation and exploitation to pursue their self-interest (Locke, 2009) and treat employees with little care or sincerity (Campbell et al., 2011). These negative behaviors can lead employees into a crisis of trust in their leaders (Hogan and Benson, 2009). Studies have shown that trust in leaders have a strong effect on employees’ OCBs (Nohe and Hertel, 2017). Thus, the lack of trust in leaders makes employees reluctant to make extra efforts that are beneficial to their leaders and their organizations (Mayer and Gavin, 2005). In addition, narcissistic leaders have low empathy and a high tendency toward exploitation (Emmons, 1984), which will lead to negative emotions, attitudes, and behaviors of employees (Li et al., 2018).

Employee OCB-L represents discretionary extra-role behaviors by which employees take the initiative to assist and support their leaders; this will produce immediate benefits to leaders and indirect contributions to the organization (Williams and Anderson, 1991; Liao and Rupp, 2005). According to self-concept theory, important individuals (such as leaders) in an organization will affect the self-conception and self-evaluation of employees (Shamir et al., 1993). Leader narcissism has the characteristics of overconfidence, exaggeration, and unreality and involves behaviors of exploiting employees, blaming, and criticizing employees for their failures (Chatterjee and Hambrick, 2007; Campbell et al., 2011). The existence of this “dark side” makes narcissistic leaders usually act as “egoists” and “dispossessors” in leader-employee interaction, and it is difficult for them to establish and maintain good interpersonal relationships with subordinates (Campbell et al., 2011). In such a work environment, over the long term, employees will tend to have a negative evaluation of, and a functional resistance toward their leaders (Li et al., 2018). They may retaliate against their leaders through supervisor-targeted counterproductive work behavior (Braun et al., 2018). Under such circumstances, it is impossible for employees to voluntarily put in extra effort to do something beneficial for the leader. In addition, narcissistic leaders can abuse their power to reinforce their own needs, and the bullying and oppression of employees can reduce employees’ self-perception (Martinez et al., 2008). In conclusion, the destructive leadership behaviors exhibited by narcissistic leaders will dampen employees’ work enthusiasm and seriously threaten their self-concepts, which will further enhance the aggression of employee behaviors and reduce civic extra-role behaviors. Therefore, we propose the following hypothesis:

*Hypothesis* 1: Leader narcissism is negatively related to employee OCB-L.

### The Mediating Role of Perceived Insider Status

Perceived insider status refers to a sense that employees have earned a “personal space” and acceptance inside their work organization (Masterson and Stamper, 2003); it reflects the extent to which an employee perceives himself or herself to be an insider within their organization (Stamper and Masterson, 2002; Dai and Chen, 2015). Chen and Aryee (2007) pointed out that perceived insider status defines employees’ identities and reflects their self-conception. According to self-concept theory, self-concept consists of the two dimensions of self-evaluation and self-conception (Dai and Chen, 2015), which are mainly derived from interaction with “significant others” (Bono and Judge, 2003). Individuals will exhibit behaviors consistent with such self-cognition. Based on this theory, narcissistic leaders, as the significant other of employees, may affect employees’ perceived insider status and thereby affect employee OCB-L.

First of all, leader narcissism means that the leader is self-centered, neglects the employees’ contributions, and may even take credit for employees’ achievements (Judge et al., 2006; Chatterjee and Hambrick, 2007). This makes the employees feel that their contributions and values are not recognized by the organization, so it is difficult for them to feel themselves to be insiders (Stamper and Masterson, 2002). Second, leaders with narcissistic personality traits are arrogant, will distrust employees’ abilities (Back et al., 2013), and will even pursue self-interests by unscrupulous means (Grijalva and Harms, 2014; Liu et al., 2017). This makes the employees feel that they are ostracized by their leaders and that their abilities are not recognized, and thus they will think of themselves as outsiders. Finally, narcissistic leaders often exhibit negative behaviors, such as bullying, hostility, denial, and criticism of employees, which damages the leader-employee exchange relationship (Haggard and Park, 2018). A high-quality leader-employee exchange relationship is an important prerequisite for employees to gain personal space and acceptance in the organization (Zhao et al., 2014). As a result, employees’ perceived insider status will be reduced. Combined with the above analysis, the egotism, arrogance, and negative behaviors of the narcissistic leader make employees feel that they are not accepted by the leader or the organization, thus reducing their perceived insider status. Therefore, we present the following hypothesis:

*Hypothesis* 2: Leader narcissism is negatively related to perceived insider status.

Perceived insider status is an important variable for predicting extra-role behavior (Stamper and Masterson, 2002; Wang and Kim, 2013). Existing studies show that higher perceived insider status means that employees have a stronger sense of belonging and greater organizational commitment (Chen and Aryee, 2007). This makes employees have a strong spirit of ownership. Therefore, when employees have a high level of perceived insider status, they will prioritize the organization’s interests. Moreover, they will not only take the initiative to finish their own work but will also complete tasks that exceed their own work requirements and are beneficial to the leaders and will engage in fewer deviant workplace behaviors (Stamper and Masterson, 2002). Conversely, when employees have a low level of perceived insider status, they will have a low level of organizational commitment and think of themselves as outsiders. It will be difficult for them to find their values in the organization, and they will often choose to show less altruistic behaviors. According to self-concept theory, employees with high perceived insider status integrate organizational membership into their self-concept (Rhoades et al., 2001), regard themselves as insiders, and act in a way that is consistent with this identity (Chen and Aryee, 2007). Therefore, we propose the following hypothesis:

*Hypothesis* 3: Perceived insider status is positively related to employee OCB-L.

According to self-concept theory, leaders can influence employees’ attitudes and behavioral tendencies through the mediating role of employees’ perceived insider status (Van Knippenberg et al., 2004). Leaders with narcissistic traits show self-concern, distrust employees, pay too much attention to themselves, and lack empathy and care for employees (Rose, 2002; Back et al., 2013). Under the influence of such a negative leadership style, employees tend to believe that their role in the organization is negligible. They perceive themselves to be ignored and difficult to be an insider. In this way, employees’ perceived insider status is reduced. They engage in behaviors consistent with this self-conception, such as silence behavior and retaliatory behavior against the organization (Carnevale et al., 2018). They are thus less likely to engage in extra-role behaviors (Stamper and Masterson, 2002). In conclusion, we propose that leader narcissism should weaken employees’ perceived insider status. This will make employees feel that they have not been recognized by their leaders and reduce their trust in and satisfaction with their leaders, thus reducing employee OCB-L. Based on the above discussion, the following hypothesis is proposed:

*Hypothesis* 4: Perceived insider status mediates the relationship between leader narcissism and employee OCB-L.

### The Moderating Role of the Need for Self-Esteem

The need for self-esteem refers to a need for “approval from others or the wish that others hold a positive view of oneself, suggesting a desire for attention and praise” (Hill, 1987, p.1009). It can be seen from this definition that the need for self-esteem reflects, to some extent, the psychological intensity of the individual’s fear of being rejected or ostracized by others. In other words, the need for self-esteem is an embodiment of feelings of self-worth, which will be affected by the attention and perceptions of others. Studies have indicated that, compared to individuals with a low need for self-esteem, individuals with a high need for self-esteem are more likely to be high self-monitors, to be competitive and to have higher levels of achievement motivation (Hill, 1987; Fuller et al., 2006). However, individuals vary in the extent to which they need self-esteem (Hill, 1987; Armeli et al., 1998). Prior studies have shown that, to some extent, employees’ need for self-esteem is hidden in their identification with their leaders and organizations and differs among different individuals (Hill, 1987; Armeli et al., 1998). Therefore, we suggest that the need for self-esteem may moderate the relationship between leader narcissism and employees’ perceived insider status.

Specifically, employees with a high need for self-esteem are more likely to care more about their leaders’ opinions of them (Fuller et al., 2006; Shen et al., 2009). As a result, they have stronger cognitive and behavioral reactions to negative evaluations and feedback. When employees with a high need for self-esteem are faced with narcissistic leaders, they are more sensitive to negative behaviors such as exploitation and suppression of employees. Thus, they will feel more ostracized by their leaders (Abrams and Hogg, 1988), and their perceived insider status will be greatly reduced. Conversely, employees with a low need for self-esteem are not afraid of rejection from others because their feelings of self-worth are based more on self-perception than on external evaluations and opinions (Fuller et al., 2006). In other words, outsiders’ opinions and evaluations are less likely to influence the extent to which employees with a low need for self-esteem tie their self-concept to the organization (Shen et al., 2009). Thus, employees with a low need for self-esteem do not care as much about rejection and negative evaluation and have a high tolerance for ostracism from the surrounding environment. Therefore, a low need for self-esteem can weaken the negative relationship between leader narcissism and perceived insider status. It can be seen that individuals with different levels of need for self-esteem have different responses to leader narcissism and then make different behavioral choices. Taking these considerations together, we propose:

*Hypothesis* 5: The need for self-esteem moderates the relationship between leader narcissism and perceived insider status such that the relationship is stronger when the need for self-esteem is high.

According to self-concept theory, once employees have a certain self-concept, they will constantly self-strengthen the concept and show corresponding attitudes and behaviors (Bono and Judge, 2003). Employees usually only accept external information that is valuable to their self-concept, and employees’ need for self-esteem often affects their value judgment of that information. Therefore, in light of the above hypothesis and theoretical derivation, we further propose a “first-stage” moderated mediation (Edwards and Lambert, 2007): the mediating effect of perceived insider status between leader narcissism and employee OCB-L is moderated by the need for self-esteem. Specifically, when employees have a high need for self-esteem, their perceived insider status is more likely to be weakened by leader narcissism, thus reducing OCB-L. By contrast, when employees have a low need for self-esteem, the influence of leader narcissism on their perceived insider status is limited, so it does not necessarily reduce OCB-L. Thus, taking hypothesis 3 and hypothesis 4 together, we propose:

*Hypothesis* 6: The need for self-esteem moderates the mediation effect of perceived insider status on the relationship between leader narcissism and employee OCB-L, such that the mediation effect is stronger when the need for self-esteem is high.

## Materials and Methods

### Sample and Procedures

Data were collected from eight small- and medium-sized enterprises in Hubei province, China. To ensure the authenticity and accuracy of the data, assurances of anonymity and confidentiality were given to respondents on the front page of the questionnaire, together with a brief outline of how their responses would be used. To decrease common method bias, data were collected in two phases, 1month apart. Matching questionnaires were distributed to leaders and employees, with each leader rating one employee. At time 1, employees filled out a questionnaire that included items measuring leader narcissism and demographic information. At this stage, a total of 210 questionnaires were sent out and 196 questionnaires were returned. One month later, at time 2, the 196 employees who completed the first-stage survey were asked to evaluate their need for self-esteem and perceived insider status. Meanwhile, their leaders reported their demographic information and rated their employees’ OCB-L. At this stage, a total of 196 questionnaires were sent out and 183 questionnaires were returned.

After incomplete and invalid questionnaires were eliminated, we finally obtained 161 valid two-stage matched leader-employee dyads, with an effective response rate of 76.7%. For leaders, 72.7% were male, with an average age of 40.2years (SD=8.74) and an average tenure in the organization of 9.44years (SD=6.80); 64.6% had a bachelor’s degree or above. For employees, 60.9% were male, with an average age of 30.8years (SD=6.99) and an average number of working years with leaders in the organization of 2.46 (SD=2.37); 64.6% have worked with their direct leaders for more than 1year, and the maximum and minimum number of working years with leaders are 15 and 0.08 respectively; 54.7% had a bachelor’s degree or above.

### Measurement of Variables

The measures in this study were originally in English. According to the suggestion of Brislin (1970), we translated the questionnaires from English to Chinese using a standard translation and back-translation procedure to ensure reliability and validity. All measures were rated on a six-point Likert scale from 1= *totally disagree* to 6= *totally agree* unless otherwise indicated.

#### Leader Narcissism

Leader narcissism was measured using a 6-item scale developed by Hochwarter and Thompson (2012). Sample items are “My leader is a very self-centered person” and “My leader has an inflated view of him/herself.” Cronbach’s alpha for this scale in this study was 0.975.

#### Perceived Insider Status

Perceived insider status was measured using a 6-item scale developed by Stamper and Masterson (2002). Sample items are “I feel very much a part of my work organization” and “My work organization makes me believe that I am included in it.” Cronbach’s alpha for this scale in this study was 0.956.

#### Organizational Citizenship Behavior Directed Toward Leader

OCB-L was measured using an 8-item scale, similar to the OCB-I (OCBs directed toward individuals) scale developed by Lee and Allen (2002). Sample items are “This employee helps me when I am absent” and “This employee willingly gives his/her time to help me when I have work-related problems.” Cronbach’s alpha for this scale in this study was 0.909.

#### Need for Self-Esteem

The need for self-esteem was measured using a 6-item scale developed by Armeli et al. (1998). Sample items are “I really want to be around people who care about who I am and what I do” and “I mainly like to be around others who think I am an important, exciting person.” Cronbach’s alpha for this scale in this study was 0.835.

#### Control Variables

We introduced several control variables into our analysis to minimize the effects of other exogenous variables. First, Owens et al. (2015) indicated that the effect of leader narcissism may vary across different leader genders or ages. Therefore, we controlled leader gender and leader age. In line with previous OCB-L research (Liao and Rupp, 2005; Li et al., 2018), we also controlled employee gender, employee age, leader and employee educational background, leader job tenure, and working years with leaders. Gender was coded as 1=male and 2=female. Educational background was coded as follows: 1=high school and below, 2=junior college, 3=bachelor’s degree, and 4=master’s degree and above.

## Analysis and Results

### Common Method Variance

Although this study used a multi-time point and multi-source method for data collection, there may still be some common method variance. Since leader narcissism, perceived insider status and need for self-esteem were measured with the same source, this study used the Harman single factor test to perform exploratory factor analysis for all items of the three variables in SPSS 22 (Podsakoff and Organ, 1986; Li et al., 2018). The exploratory factor analysis was used to extract a principal component, and the results show that the cumulative interpretation variance percentage of the first factor is 41.697%, which is lower than 50% (Podsakoff and Organ, 1986). Therefore, it can be concluded that there is no serious problem of common method variance in this study.

### Confirmatory Factor Analysis

We used Mplus 7.4 to perform confirmatory factor analyses to verify discriminant validity among variables in this study. The measurement model contained four concepts (i.e., leader narcissism, perceived insider status, need for self-esteem, and OCB-L) and 26 items. As Table 1 showed, the four-factor model fit the data better (*χ*^2^=53.016, *df*=48, *χ*^2^/*df*=1.105, CFI=0.998, TLI=0.997, RMSEA=0.025, SRMR=0.024) than three-factor model (*χ*^2^=318.549, df=51, *χ*^2^/*df*=6.246, CFI=0.870, TLI=0.832, RMSEA=0.181, SRMR=0.115), two-factor model (*χ*^2^=740.385, *df*=53, *χ*^2^/df=13.970, CFI=0.666, TLI=0.584, RMSEA=0.284, SRMR=0.190; *χ*^2^=860.346, *df*=53, *χ*^2^/*df*=16.233, CFI=0.608, TLI=0.512, RMSEA=0.308, SRMR=0.204), and one-factor model (*χ*^2^=1033.843, *df*=54, *χ*^2^/df=19.145, CFI=0.524, TLI=0.419, RMSEA=0.336, SRMR=0.230), indicating that the overall scale had better discriminative validity. In addition, we calculated the composite reliability (CR) and average variance extracted (AVE) of each variable. As shown in Table 2, the CRs of all variables ranged from 0.882 to 0.980 and all were above 0.70; the AVEs of all variables ranged from 0.566 to 0.890 and all were above 0.50 (Fornell and Larcker, 1981). The results showed that the convergent validity of each scale was better.

**Table 1:** Confirmatory factor analyses.

Models	*χ* ^2^	*df*	*χ*^2^/*df*	CFI	TLI	RMSEA	SRMR
Four-factor model (LN; PIS; OCB-L; NFSE)	53.016	48	1.105	0.998	0.997	0.025	0.024
Three-factor model (LN; PIS+OCB-L; NFSE)	318.549	51	6.246	0.870	0.832	0.181	0.115
Two-factor model (LN+PIS+ NFSE; OCB-L)	740.385	53	13.970	0.666	0.584	0.284	0.190
Two-factor model (LN+PIS+OCB-L; NFSE)	860.346	53	16.233	0.608	0.512	0.308	0.204
One-factor model (LN+PIS+OCB-L+NFSE)	1033.843	54	19.145	0.524	0.419	0.336	0.230

LN, leader narcissism; PIS, perceived insider status; and NFSE, need for self-esteem.

**Table 2:** Means, SDs, and intercorrelations of variables.

Variables	1	2	3	4	5	6	7	8	9	10	11	12
1.E-Gen	−											
2.E-Age	0.105											
3.E-Edu	0.004	−0.321^***^										
4.E-WYWL	0.069	0.250^**^	0.011									
5.L-Gen	−0.006	−0.072	0.120	0.083								
6.L-Age	0.029	0.053	0.096	0.012	−0.064							
7.L-Edu	−0.155	−0.066	−0.108	−0.162^*^	0.205^**^	−0.010						
8.L-JT	0.017	−0.127	0.230^**^	0.120	0.025	0.395^***^	−0.231^**^					
9.LN	0.097	0.151	−0.137	−0.109	0.018	−0.071	0.004	−0.066	**(0.890)**			
10.PIS	−0.016	0.019	0.006	−0.038	−0.016	0.018	−0.055	−0.066	−0.422^***^	**(0.823)**		
11.OCB-L	0.088	−0.082	−0.044	−0.057	−0.074	0.100	−0.012	0.124	−0.170^*^	0.369^***^	**(0.622)**	
12.NFSE	−0.040	0.030	−0.042	−0.064	0.039	0.095	0.042	0.143	−0.169^*^	0.125	0.081	**(0.566)**
M	1.391	30.814	2.360	2.462	1.273	40.173	2.901	9.438	4.078	2.928	3.035	4.844
SD	0.490	6.988	0.952	2.375	0.447	8.737	1.050	6.804	1.088	1.022	0.789	0.854
CR									0.980	0.965	0.923	0.882

1=Male, 2=Female. E-Gen, employee gender; E-Age, employee age; E-Edu, employee education; E-WYWL, employees’ working years with leaders, L-Gen, leader gender; L-Age, leader age; L-Edu, leader education; L- JT, leader job tenure; LN, leader narcissism; PIS, perceived insider status; and NFSE, need for self-esteem. Bracketed bold values on the diagonal are the AVE of each scale.

* *p*<0.05;

** *p*<0.01;

*** *p*<0.001.

### Descriptive Statistics and Correlations

Table 2 presented the means, SDs, CR, AVE, and correlations for all variables in our study. As shown in Table 2, leader narcissism was significantly and negatively correlated with employee OCB-L (*r*=−0.170, *p*<0.05) and perceived insider status (*r*=−0.422, *p*<0.001), which initially verified Hypothesis 1 and Hypothesis 2. Meanwhile, perceived insider status was positively correlated with employee OCB-L (*r*=0.369, *p*<0.001), which initially verified Hypothesis 3. These results provided preliminary support for subsequent hypothesis testing.

### Hypothesis Testing

This study conducted hierarchical regression analysis to test the research hypotheses using SPSS 22 software, as shown in Table 3. We first entered all control variables into the model (Model 1) and then added leader narcissism into the model (Model 2). Model 2 showed that after controlling demographic variables, leader narcissism had a significantly negative impact on perceived insider status (*β*=−0.458, *p* <0.001), supporting hypothesis 2. Similarly, on the basis of Model 4, we added leader narcissism into the model (Model 5). Model 5 showed that leader narcissism had a significantly negative impact on employee OCB-L (*β*=−0.181, *p*<0.05). Hence, hypothesis 1 was supported. Moreover, Model 6 showed that after controlling demographic variables, perceived insider status had a significantly positive impact on employee OCB-L (*β*=0.385, *p*<0.001), supporting hypothesis 3. Comparing Model 5 and Model 7, after the introduction of perceived insider status, the coefficient of the impact of leader narcissism on employee OCB-L was decreased and insignificant (*β*=−0.181, *p*<0.05, Model 5; *β*=−0.006, n.s., Model 7). Meanwhile, perceived insider status still had a significantly positive impact on employee OCB-L (*β*=0.382, *p*<0.001). The results revealed that perceived insider status had a complete mediating effect between leader narcissism and employee OCB-L. Hypothesis 4 was supported. To verify the robustness of the mediating effect, we used Model 4 in the PROCESS macro of Hayes (2013) to verify the results. Based on 5,000 bootstrapping samples, the results showed that the indirect effect of leader narcissism on employee OCB-L *via* perceived insider status was significant (indirect effect=−0.127, 95% CI=[−0.211, −0.056], excluding 0), thus hypothesis 4 was further supported.

**Table 3:** Regression analysis of hypotheses.

Variables	Perceived insider status			Employee OCB-L			
	Model 1	Model 2	Model 3	Model 4	Model 5	Model 6	Model 7
Employee gender	−0.028	0.013	0.009	0.101	0.118	0.112	0.113
Employee age	0.018	0.093	0.097	−0.106	−0.077	−0.113	−0.112
Employee education	0.020	−0.020	−0.007	−0.101	−0.117	−0.108	−0.109
Employees’ working years with leaders	−0.044	−0.119	−0.078	−0.043	−0.072	−0.026	−0.027
Leader gender	0.012	0.035	0.023	−0.068	−0.059	−0.073	−0.073
Leader age	0.058	0.023	0.015	0.062	0.048	0.040	0.039
Leader education	−0.090	−0.096	−0.081	0.021	0.019	0.056	0.056
Leader job tenure	−0.106	−0.098	−0.120	0.119	0.123	0.160	0.160
Leader narcissism		−0.458^***^	−0.425^***^		−0.181^*^		−0.006
Need for self-esteem			0.116				
Leader narcissism * Need for self-esteem			−0.182^*^				
Perceived insider status						0.385^***^	0.382^***^
F	0.297	4.478^***^	4.319^***^	1.005	1.472	4.087^***^	3.654^***^
R^2^	0.015	0.211^***^	0.242^*^	0.050	0.081^*^	0.196^***^	0.196^***^
△R^2^		0.195^***^	0.028^*^		0.030^*^	0.146^***^	0.115^***^

*All regression coefficients are standard regression coefficients*.

* *p*<0.05;

*** *p*<0.001.

**Figure 2:**
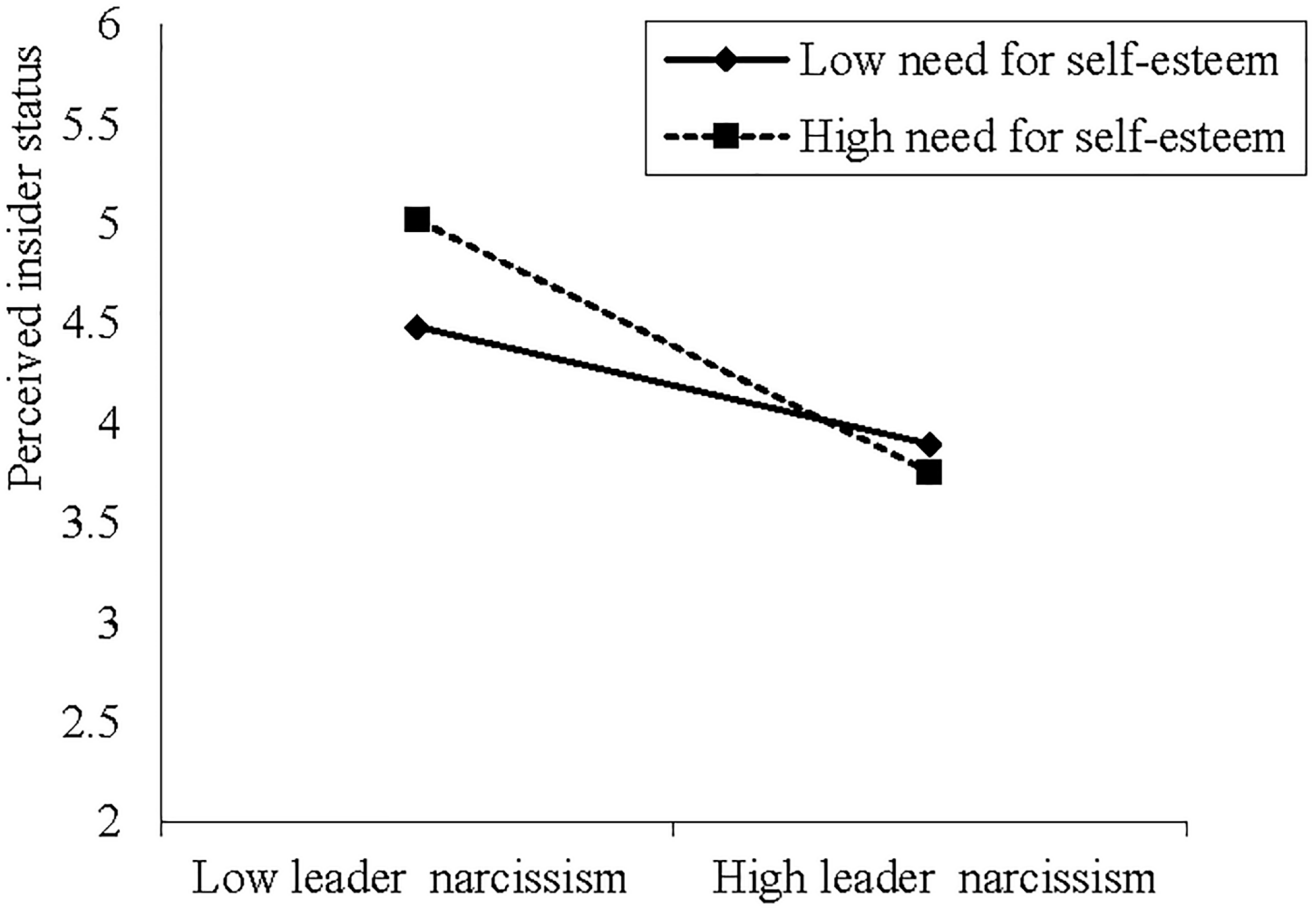
Moderating effect of need for self-esteem on relationship between leader narcissism and perceived insider status.

We conducted a three-step hierarchical regression analysis to test the moderation effect of the need for self-esteem. To reduce multicollinearity problems, we first centralized the independent variable and moderator variable before forming the interaction term according to the method of Aiken and West (1991). As shown in Model 3 in Table 3, the interaction between leader narcissism and the need for self-esteem was significantly and negatively related to perceived insider status (*β*=−0.182, *p*<0.05). Hypothesis 5 was supported. Simple slope tests were also conducted to further verify the interpretation of this interaction (Aiken and West, 1991). Figure 2 plotted the relationship between leader narcissism and perceived insider status at one SD above and below the mean of the need for self-esteem. The results showed that for employees with a high need for self-esteem (one SD above the mean), the negative relationship between leader narcissism and perceived insider status was stronger (*β*=−0.541, *p*<0.001). Conversely, for employees with a low need for self-esteem (one SD below the mean), the negative relationship between leader narcissism and perceived insider status was weaker and not significant (*β*=−0.200, n.s.). These results provided additional support for hypothesis 5.

We used Model 7 in the PROCESS macro of Hayes (2013) to test the moderated mediation effect, as shown in Table 4. Results from 5,000 times bootstrapping showed that when employees had a low need for self-esteem, leader narcissism did not have a significant indirect effect on employee OBC-L *via* perceived insider status (indirect effect=−0.068, 95% CI=[−0.147, 0.009], including 0). In contrast, when employees had a high need for self-esteem, leader narcissism had a significant indirect effect on employee OBC-L *via* perceived insider status (indirect effect=−0.168, 95% CI=[−0.277, −0.077], excluding 0). Moreover, the index of moderated mediation was significant (indirect effect=−0.118, 95% CI=[−0.197, −0.054], excluding 0). Together, the results showed that the indirect effect of leader narcissism on employee OBC-L through the mediating role of perceived insider status would be stronger when the need for self-esteem was high. Thus, hypothesis 6 was supported.

**Table 4:** Results of the moderated mediation.

Need for self-esteem	Leader narcissism → perceived insider status → employee OCB-L		
	Indirect effect	SE	95% CI
Low (mean−1SD)	−0.068	0.039	[−0.147, 0.009]
Mean	−0.118	0.037	[−0.197, −0.054]
High (mean+1SD)	−0.168	0.051	[−0.277, −0.077]

## Discussion

The present study investigated how leader narcissism affects employee OCB-L through the mediating effect of perceived insider status. We found that leader narcissism had a significantly negative influence on employee OCB-L. Perceived insider status played a full mediating role in the impact of leader narcissism on employee OCB-L. Furthermore, we found that the need for self-esteem could strengthen the effect of leader narcissism on employee OCB-L *via* perceived insider status. As our results showed, the mediating effect of perceived insider status on the relationship between leader narcissism and employee OCB-L was stronger for employees with a high need for self-esteem than for employees with a low need for self-esteem.

### Theoretical Implications

Our study makes several theoretical contributions. First, this study explored and tested the negative impact of leader narcissism on employee OCB-L, which enriched our understanding of the outcome variables of leader narcissism in the Chinese context. Prior studies have explored the relationship between leader narcissism and employees’ workplace behaviors, such as helping behavior (Carnevale et al., 2018), voice behavior (Liao et al., 2019b; Huang et al., 2020; Yao et al., 2020), innovation behavior (Yang et al., 2020; Norouzinik et al., 2021), and proactive behavior (Liao et al., 2019a). However, these employees’ workplace behaviors are either intended to help their colleagues or to promote organizational development and improvement. By contrast, these studies have neglected leader-directed behaviors. Because employee OCB-L can meet narcissistic leaders’ unique needs (Gerstner et al., 2013) and is good for forming a benign interaction between leaders and employees (Li et al., 2018), so it is of great importance to explore how leader narcissism affects employee OCB-L. Our results indicate that leader narcissism will reduce employee OCB-L. This study enriches and supplements prior researches on the dark side influence of leader narcissism and contributes to the literature on leader narcissism and employees’ followership behavior.

Second, this study proposed and tested the mediating role of perceived insider status between leader narcissism and employee OCB-L and revealed the internal mechanism underlying that relationship. By reviewing the prior literatures on the effects of leader narcissism on employees’ behaviors, our study concluded that most scholars have mainly followed three perspectives: the social exchange perspective (O’Boyle et al., 2012; Wang et al., 2018; Liao et al., 2019b), the conservation of resources perspective (Huang et al., 2020; Yao et al., 2020; Norouzinik et al., 2021) and the social cognitive perspective (Zhang et al., 2018; Aboramadan et al., 2021). Although existing studies have helped us to understand the influencing mechanism of leader narcissism on employees’ behaviors from different perspectives, no research to date has explored it from the perspective of self-concept. Our results show that employees’ perceived insider status plays a full mediating role in the impact of leader narcissism on employee OCB-L, providing an explanatory mechanism for the influencing effect of leader narcissism from the self-concept perspective. Therefore, this study not only expands the research perspective of the leader narcissism literature but also enriches our understanding of the internal mechanism of the influence of leader narcissism on employee OCB-L in the organizational context.

Finally, this study examined and confirmed the moderating role of the need for self-esteem and revealed the boundary conditions under which leader narcissism influences employee OCB-L through perceived insider status. Based on the perspective of leaders, many studies have shown that leader humility (Owens et al., 2015), leader consultation (Carnevale et al., 2018), and leader effectiveness (Liu et al., 2021) can mitigate the harmful effects of leader narcissism on employees’ behaviors and job performance. However, few studies have explored whether the employees’ need for self-esteem has a contingency effect on leader narcissism. Because employees have different individual characteristics, the effectiveness of the influence of leadership often varies from person to person. By introducing the need for self-esteem as a moderator variable, our results show that a high need for self-esteem strengthens the negative effect of leader narcissism on perceived insider status and enhances the indirect effect of perceived insider status on leader narcissism and employee OCB-L. This further suggests that employees’ traits play a critical role in determining how employees experience leader narcissism. This study deepens our understanding of the complexities underlying employees’ reactions toward leader narcissism and provides a new way for employees to deal with leader narcissism.

### Managerial Implications

Our study has significant implications for managerial practices. First, our results show that leader narcissism negatively affects employee OCB-L. Therefore, organizations and leaders should take effective measures to prevent the negative effects of leader narcissism. On the one hand, organizations should prevent leaders from narcissism at the source. For example, narcissistic personality tests can be used in the selection and promotion of leaders to identify candidates with high levels of narcissism and negative traits. Organizations can evaluate and supervise the use of leaders’ power and reduce the possibility of narcissistic leaders abusing their power for personal gain. Humility is an interpersonal and epistemic stance (D’Errico, 2020), which plays an important role in crisis management (Scardigno et al., 2021) and has a tempering effect on narcissism (Owens et al., 2015). Thus, organizations should provide leadership behavior training courses and promote humble leadership behavior. On the other hand, leaders should face up to their own characteristics, correct their attitudes, words, and deeds as leaders, seek advantages and avoid disadvantages, and restrain their narcissism. Moreover, in the management process, leaders should never forget their original intention, be modest and prudent, and trust, support, and affirm the abilities and contributions of employees.

Second, our results show that leader narcissism negatively affects employee OCB-L through the mediating role of perceived insider status. That is to say, when employees perceive themselves to be insiders of the organization, they will show more OCB-L. Therefore, organizations should use specific human resource management initiatives to enhance employees’ identity recognition as insiders of the enterprise. Enterprise leaders should try to convey supportive signals to employees through human resource management policies and actions, such as providing skills training and encouraging employees to participate in decision-making. They should also implement all policies and regulations fairly, treat every employee equally, show more care and trust for employees, and give employees more autonomy and flexibility in their work. In a word, effective measures should be taken to make employees feel valued and recognized by leaders and the organization and to enhance perceived insider status, so as to foster more OCB-L.

Finally, our results show that the need for self-esteem can act as a moderator for leader narcissism’s negative effects on perceived insider status and OCB-L. In other words, the higher the need for self-esteem, the stronger the negative impact of leader narcissism on OCB-L through perceived insider status. Therefore, leaders should implement differentiated management for employees and give more trust and positive evaluations to employees with a high need for self-esteem. These actions will mitigate the negative impact of leader narcissism on perceived insider status. In addition, our results indicate that a low need for self-esteem can alleviate the negative impact of leader narcissism on employee OCB-L *via* perceived insider status. It means that employees with a low need for self-esteem deal with narcissistic leaders effectively. Therefore, organization should pay attention to understand the level of employees’ need for self-esteem to better realize leader-member fit and generate positive interactions.

### Limitations and Future Research

Although this study makes theoretical contributions and has practical implications, our study also has several limitations that should be explored in future research. First, this study was conducted in China, a culture characterized by a highly collectivistic orientation and high power distance (House et al., 2004). It is questionable, therefore, whether its findings can be generalized to other cultures. For example, in low power distance cultures, employees may have different understandings of leader narcissism and its influence would be different. Hence, future studies could test our theoretical model in different cultures to achieve a more comprehensive understanding of leader narcissism. Second, although we collected leader-employee dyadic data at two time points, causality could not be unambiguously established. Hence, future studies could use an experimental design to better test the causal relationships among the variables we studied. Finally, this study only focused on the moderating effect of the need for self-esteem at the individual level of employees, while the relationship between leader narcissism and perceived insider status may be moderated by many factors at the leadership level and the organizational level. Hence, future studies can build a multi-level model for further exploration.

## Conclusion

The relationship between leader narcissism and employees’ work behavior has attracted extensive attention from scholars. Our study results deepen our understanding of how leader narcissism affects employee OCB-L, a leader-directed followership behavior. Specifically, we have found that leader narcissism is negatively related to employees’ perceived insider status and OCB-L. Perceived insider status completely mediates the negative correlation between leader narcissism and employee OCB-L. The need for self-esteem moderates the negative effect of leader narcissism on perceived insider status and the mediating effect of perceived insider status between leader narcissism and employee OCB-L. These findings answer, from the perspective of self-concept, the question of how leader narcissism influences employee OCB-L *via* perceived insider status.

## Data Availability Statement

The raw data supporting the conclusions of this article will be made available by the authors, without undue reservation.

## Author Contributions

HW designed the research and revised the whole paper. GL analyzed the data and wrote and revised the paper. MW and YD collected the data and discussed the results. All authors contributed to the article and approved the submitted version.

## Funding

This research was supported by the Program for the Humanity and Social Science Youth Foundation of the Ministry of Education of China (19YJC630165).

## Conflict of Interest

The authors declare that the research was conducted in the absence of any commercial or financial relationships that could be construed as a potential conflict of interest.

## Publisher’s Note

All claims expressed in this article are solely those of the authors and do not necessarily represent those of their affiliated organizations, or those of the publisher, the editors and the reviewers. Any product that may be evaluated in this article, or claim that may be made by its manufacturer, is not guaranteed or endorsed by the publisher.
